# Morphometric analysis of tumor microvessels for detection of hepatocellular carcinoma using contrast-free ultrasound imaging: A feasibility study

**DOI:** 10.3389/fonc.2023.1121664

**Published:** 2023-04-13

**Authors:** Soroosh Sabeti, Redouane Ternifi, Nicholas B. Larson, Michael C. Olson, Thomas D. Atwell, Mostafa Fatemi, Azra Alizad

**Affiliations:** ^1^ Department of Physiology and Biomedical Engineering, Mayo Clinic College of Medicine and Science, Rochester, MN, United States; ^2^ Department of Quantitative Health Sciences, Mayo Clinic College of Medicine and Science, Rochester, MN, United States; ^3^ Department of Radiology, Mayo Clinic College of Medicine and Science, Rochester, MN, United States

**Keywords:** angiogenesis, flow imaging, hepatocellular carcinoma, liver tumors, contrast-free ultrasound

## Abstract

**Introduction:**

A contrast-free ultrasound microvasculature imaging technique was evaluated in this study to determine whether extracting morphological features of the vascular networks in hepatic lesions can be beneficial in differentiating benign and malignant tumors (hepatocellular carcinoma (HCC) in particular).

**Methods:**

A total of 29 lesions from 22 patients were included in this work. A post-processing algorithm consisting of clutter filtering, denoising, and vessel enhancement steps was implemented on ultrasound data to visualize microvessel structures. These structures were then further characterized and quantified through additional image processing. A total of nine morphological metrics were examined to compare different groups of lesions. A two-sided Wilcoxon rank sum test was used for statistical analysis.

**Results:**

In the malignant versus benign comparison, six of the metrics manifested statistical significance. Comparing only HCC cases with the benign, only three of the metrics were significantly different. No statistically significant distinction was observed between different malignancies (HCC versus cholangiocarcinoma and metastatic adenocarcinoma) for any of the metrics.

**Discussion:**

Obtained results suggest that designing predictive models based on such morphological characteristics on a larger sample size may prove helpful in differentiating benign from malignant liver masses.

## 1 Introduction

Hepatocellular carcinoma (HCC) is the most frequently occurring malignancy in the liver, accounting for approximately 70 to 90 percent of all primary liver cancers (1, 2). In the year 2020, primary liver cancer ranked third among all cancers in terms of mortality rate worldwide (3). As in most other cancer types, early diagnosis of HCC is extremely important in implementation of timely intervention and determination of proper treatments (4). Hindering HCC progression in early stages can lead to higher survival rate and lower probability of postoperative recurrence (5–7). Detection of the presence of liver lesions is typically an important step in potential HCC diagnosis while screening patients with liver disorders and abnormalities (8, 9).

HCC often develops in patients with underlying liver disease (10). Viral hepatitis, autoimmune liver disorders, hemochromatosis, and nonalcoholic steatohepatitis (NASH) are among diseases which can cause chronic damage to the liver resulting in cirrhosis, and in some cases lead directly to the development of HCC (11). Thus, regular surveillance of such patients is of high importance. Different imaging techniques, including ultrasound (US), computed tomography (CT), and magnetic resonance imaging (MRI), are often used for accurate diagnosis of HCC (12). Even though research suggests inferior performance of US (especially without the use of contrast agents) in comparison with CT and MRI in detecting HCC (13, 14), US imaging is generally the method of choice for frequent routine screening due to its accessibility, portability, ease of operation, cost-effectiveness and nonionizing nature (15–17).

Angiogenesis, or the formation of new blood vessels, is a common occurrence in the development and growth of neoplasms (18, 19). HCCs are known to exhibit hypervascularity (20, 21), and therefore, identification and characterization of vascular structures in liver masses can help in establishing biomarkers resulting in more accurate diagnoses. MRI, and CT have been employed in several studies to investigate blood flow and vasculature in HCC (22–25). The use of US imaging (with and without contrast-enhancement) for characterization and quantification of vascularity and hemodynamics in liver lesions has been reported in a number of studies (26–40). In particular, Yang et al. (41) utilized the ultrasound-based technique of superb microvascular imaging (SMI) to visualize microvascular structure in liver lesions and demonstrated its diagnostic capability with respect to HCC. Oezdemir et al. (42) presented a contrast-enhanced ultrasound (CEUS) data processing method for vasculature analysis and investigated the efficacy of using morphologic characteristics of HCC vascular networks in predicting the response to transarterial chemoembolization.

In this paper, we utilize a previously introduced (43) technique for visualization of microvasculature networks obtained from contrast-free ultrasound imaging data. Since this method is shown to be capable of visualizing sub-millimeter vessels, as small as 300 µm, it has been termed high-definition microvasculature imaging (HDMI). The proposed quantitative HDMI is equipped with a series of image processing operations, morphological filtering and vessel enhancement to extract and quantify vessel morphological parameters as quantitative vessel biomarkers (43–45). This method has been used for differentiation of breast masses (45–47). The present work constitutes an evaluation framework to distinguish HCCs from benign lesions. We hypothesize, by providing information regarding tumor vessel morphological features as quantitative biomarkers, the proposed contrast-free HDMI has the potential to objectively classify early HCC from benign liver lesions, thus rendering this method operator independent and eliminating the observer/reader variability for a reliable clinical use. Statistical analysis shows the potential of this framework in accurate characterization of malignant tumors.

## 2 Materials and methods

### 2.1 Participants

**Figure 1:**
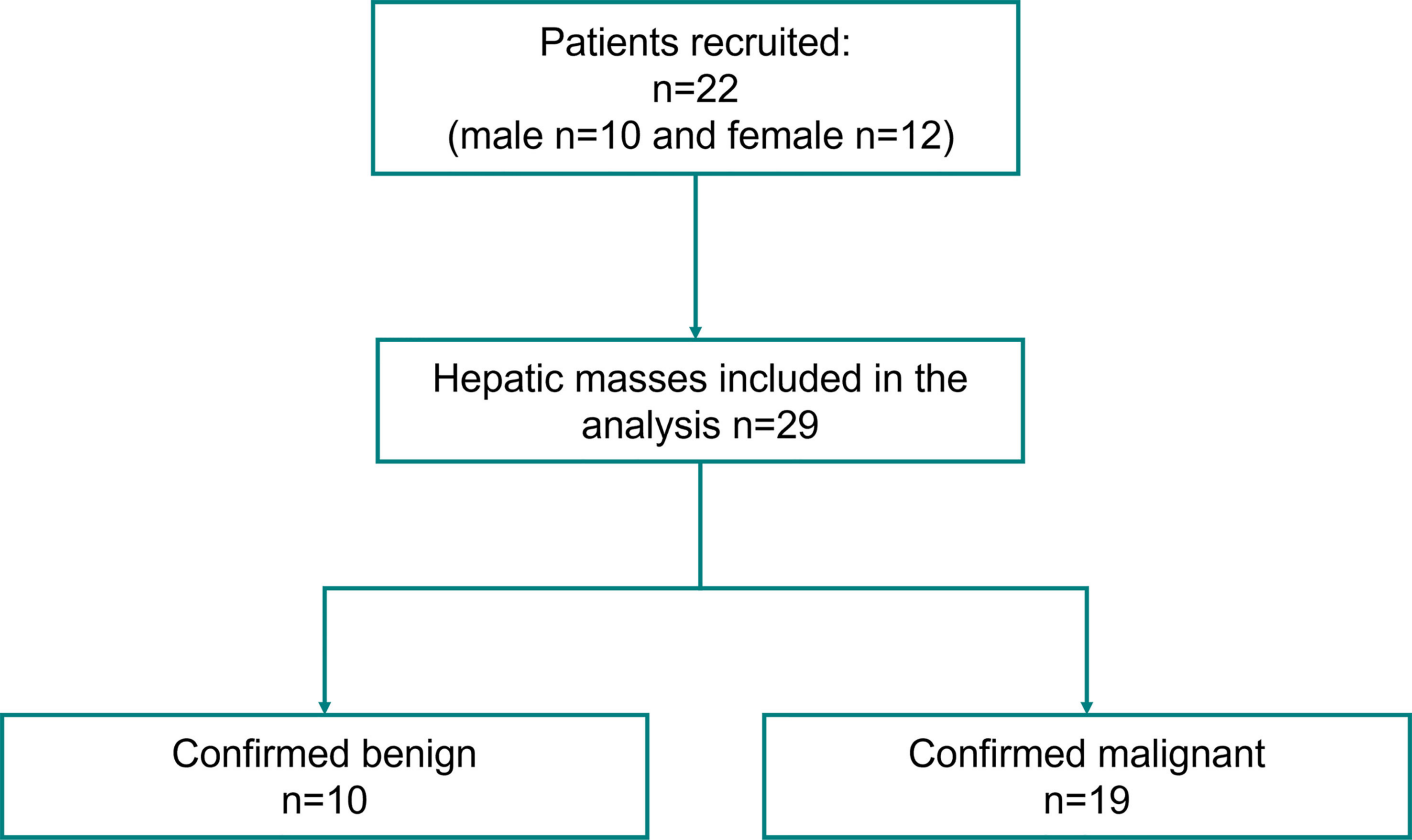
Participants flowchart.

This prospective study, which was conducted from August 2020 to September 2021, received institutional review board approval (IRB#: 16-009435) and was in compliance with the Health Insurance Portability and Accountability Act. A signed written informed consent with permission for publication was obtained from each enrolled participant prior to the study. A total of 22 patients, ages ranged 19 - 82 years, mean age 61.6 ± 16.2, with 29 identified hepatic mass/masses on their screening ultrasound imaging, were enrolled in this study. Details of participant selection are provided in **Figure 1**. Patients were selected based on the criteria of having suspicious hepatic lesions and clinical diagnoses through pathology or cross-sectional imaging were used as the gold standard for creating the classes and labels.

### 2.2 Microvessel image acquisition and generation

For each participant, the ultrasound examinations were performed by one of two trained sonographers, with more than 35 and 15 years of experience, respectively. Subjects were scanned using an ultrasound system, Alpinion E-Cube 12R ultrasound machine (Alpinion Medical Systems, Seoul, South Korea), using a curved array transducer C1-6 operating at 3.6 MHz frequency. This system is capable of plane wave imaging and provides high-frame-rate images in the form of raw in-phase and quadrature (IQ) beamformed data for a total duration of 3 seconds, forming each frame of the data using 5-angle coherent plane wave compounding (48). For image acquisition, patients were instructed to stay still and halt respiration for approximately 3 seconds during data acquisition to reduce motion artifacts. To increase reproducibility, at least 2 acquisitions at each scan orientation were acquired. The methods for obtaining HDMI images, vessel extraction and steps for vessel segmentation have been detailed in (43, 44), as well as **Supplementary Material 1**.

### 2.3 Quantification of morphological parameters of tumor microvessels

For each case, the lesion of interest was manually segmented with the help of an experienced sonographer, to enable vessel quantification inside and outside the lesion separately. The output of the segmentation would then constitute a binary mask, delineating the region of interest (ROI). This mask underwent a 10mm dilation to include perilesional vascularity. This 10mm dilation was applied consistently to all of the masks ensuring sufficient area in the periphery of all lesions (regardless of their size) is included in the analysis. In some cases, where the lesions were large, or were situated in the proximity of the edges of the scanning region, the dilated mask would extend beyond the boundaries of the image, in which case we would use as much information as available within the image for quantification. The resulting mask was subsequently applied to the microvasculature image to limit vessel quantification within the ROI.

Vessel quantification procedures were implemented on the ROI beginning by binarizing the image using an empirically chosen threshold to ensure an adequate balance between background noise exclusion and keeping the vascular structure intact. Several morphological operations were then employed to remove small objects that do not correspond to actual vessels, as well as to fill the spurious holes inside vascular structures that might appear as a result of the visualization filtering processes. Next, a thinning algorithm was applied to the resulting binary image to extract the centerline of the vascular networks, to obtain what is referred to as the skeleton image.

Vessel quantification operations were performed on the skeleton image in order to compute discriminating metrics that would separate benign lesions from malignant ones. In this paper, we have used nine metrics that characterize the morphology and complexity of the vascular structures inside hepatic lesions. These metrics include: vessel density (*VD*), number of vessel segments (*NV*), number of branch points (*NB*), mean tortuosity as measured by the distance metric (*τ_mean_*), maximum tortuosity as measured by the distance metric (*τ_max_*), mean diameter (*D_mean_*), fractal dimension (FD), mean of Murray’s deviation (*MD_mean_*), mean bifurcation angle (*BA_mean_*). More details on the quantification procedure and the computation of these morphological features can be found in (44, 45), as well as **Supplementary Material 1**.

### 2.4 Statistical analysis

Quantitative variables are summarized as mean ± standard deviation (SD). To evaluate the discrimination of the metrics in differentiating the tumors, two separate analyses were performed. First, the lesions were divided into two groups, benign lesions and HCC lesions. A two-sided Wilcoxon rank sum test was applied to test for differences in distributions of the metric values for the two groups and corresponding p-values were obtained. Next, a new set was created by adding the other malignant lesions (cholangiocarcinoma (CCA) and metastatic adenocarcinoma) to the HCC cases, this time with the objective of separating benign masses from malignant ones. In all cases, p-values less than 0.05 were declared as statistically significant. All data processing and analyses were performed in MATLAB R2019a (The Mathworks Inc., Natick, MA, USA).

## 3 Results

A total of 22 enrolled patients, consisting of 10 males and 12 females (ranging in age from 19 to 82 years, mean age 61.6 ± 16.2 years), with 29 hepatic masses (10 benign, 19 malignant) were examined by quantitative HDMI. The size of these lesions (diameter across the largest axial/lateral dimension) ranged from 16.8mm to 150.3mm with a mean diameter 45.7 ± 29.3mm. Clinical diagnoses (pathological or through cross-sectional imaging) were used to label the lesions. Among the 29 lesions, 27 ultimately underwent needle biopsy for pathologic diagnosis and 2 were radiologically confirmed based on typical contrast-enhanced CT and/or MRI imaging appearance. The 19 malignant cases included 11 hepatocellular carcinoma, 7 cholangiocarcinoma, and 1 metastatic adenocarcinoma of pancreatic origin. The 10 benign hepatic masses included 6 pathologically proven hepatocellular adenoma, 2 atypical hemangioma and 2 LI-RADS 3A labeled masses. **Table 1** summarizes the lesion information included in this study.

**Table 1:** Summary of the lesion information.

Total patients	22
Female	12
Male	10
Total lesions	29
Mass size (diameter in largest dimension)^a^	[16.8mm,150.3mm] (45.7 ± 29.3mm)
Lesion types	
Benign	10
Hepatocellular adenoma	6
Atypical hemangioma	2
LI-RADS 3A	2
Malignant	19
HCC	11
Intrahepatic cholangiocarcinomas (CCA)	7
Metastatic adenocarcinoma with pancreatic origin	1

a Numbers in the brackets show the minimum and the maximum diameter. The mean ± standard deviation of the diameters is presented in parentheses.

### 3.1 Microvessel visualization and quantification for different groups of liver masses

In this section we present the visual comparison along with metric values of quantitative HDMI, for three groups of malignant and benign liver masses: 1) two larger malignant liver masses (HCC and CCA), 2) two smaller deep-seated liver masses (HCC and benign), and 3) two very small benign and malignant liver masses.

**Figure 2:**
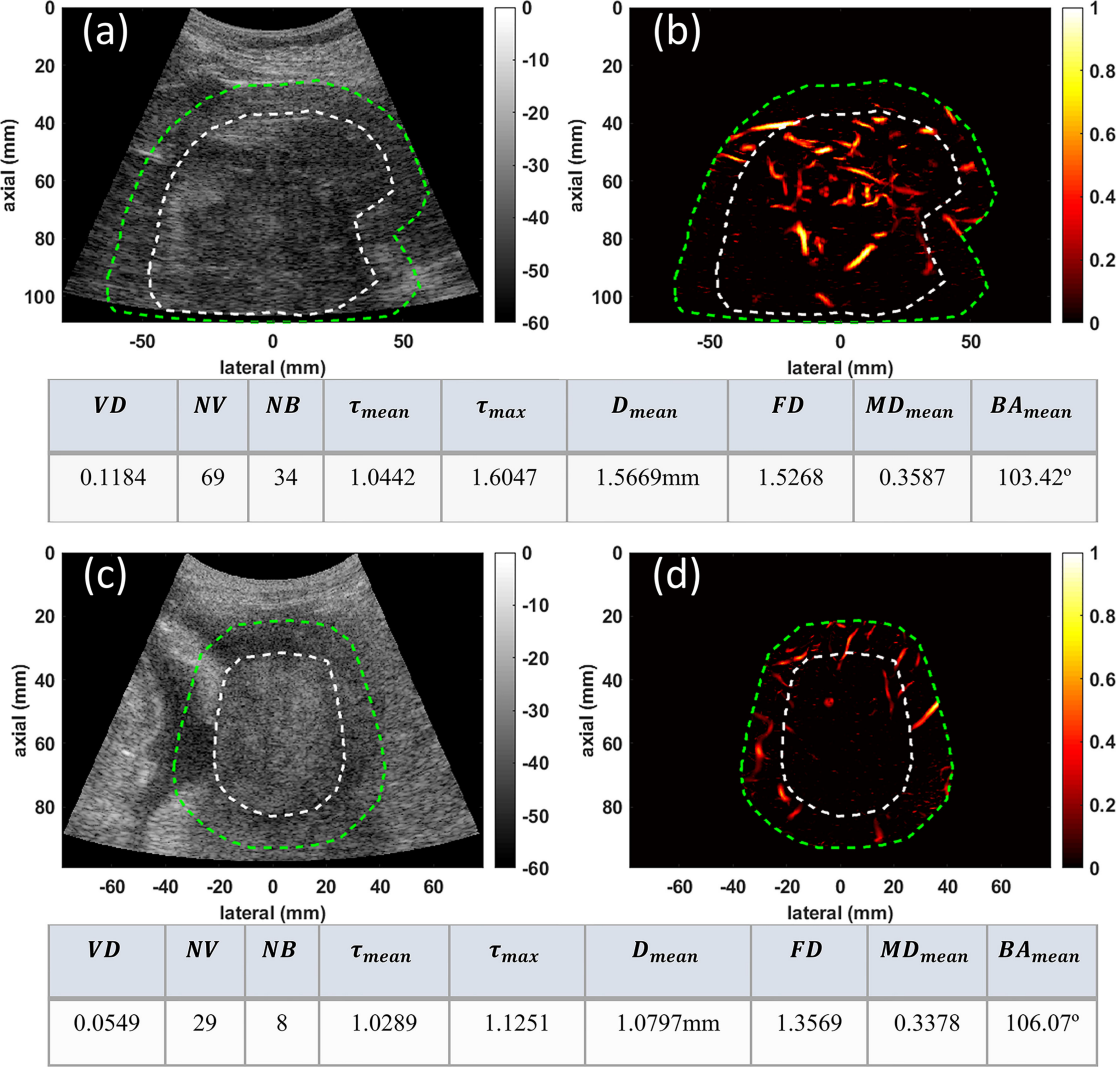
Examples of visualization and quantification results for two malignant lesions. **(A)** B-mode image of a 94mm HCC tumor **(B)** Microvasculature image inside the dilated HCC tumor mask **(C)** B-mode image of a 51mm CCA lesion **(D)** Microvasculature image inside the dilated CCA tumor mask. In all images the white dashed line indicates the boundaries of the lesion, and the green dashed line delineates the boundaries after a 10mm dilation. The tables below each row of images show metric values for the HCC (top) and CCA (bottom) tumors.

In group one, the conventional B-mode ultrasound and HDMI images of two larger malignant liver masses, HCC and CCA, respectively measuring 94mm and 51mm in the largest axial/lateral dimension, are shown for visual comparison in **Figure 2**. Microvasculature image inside the 10mm dilated mask for HCC (**Figure 2B**) presents more vascularity than that of CCA (**Figure 2D**). The quantified metrics presented in the tables below of each row show lower values for *VD, NV, NB, FD, MD_mean_*, *D_mean_*, *τ_mean_*, and *τ_max_* but higher value of *BA_mean_* in CCA compared to HCC.

**Figure 3:**
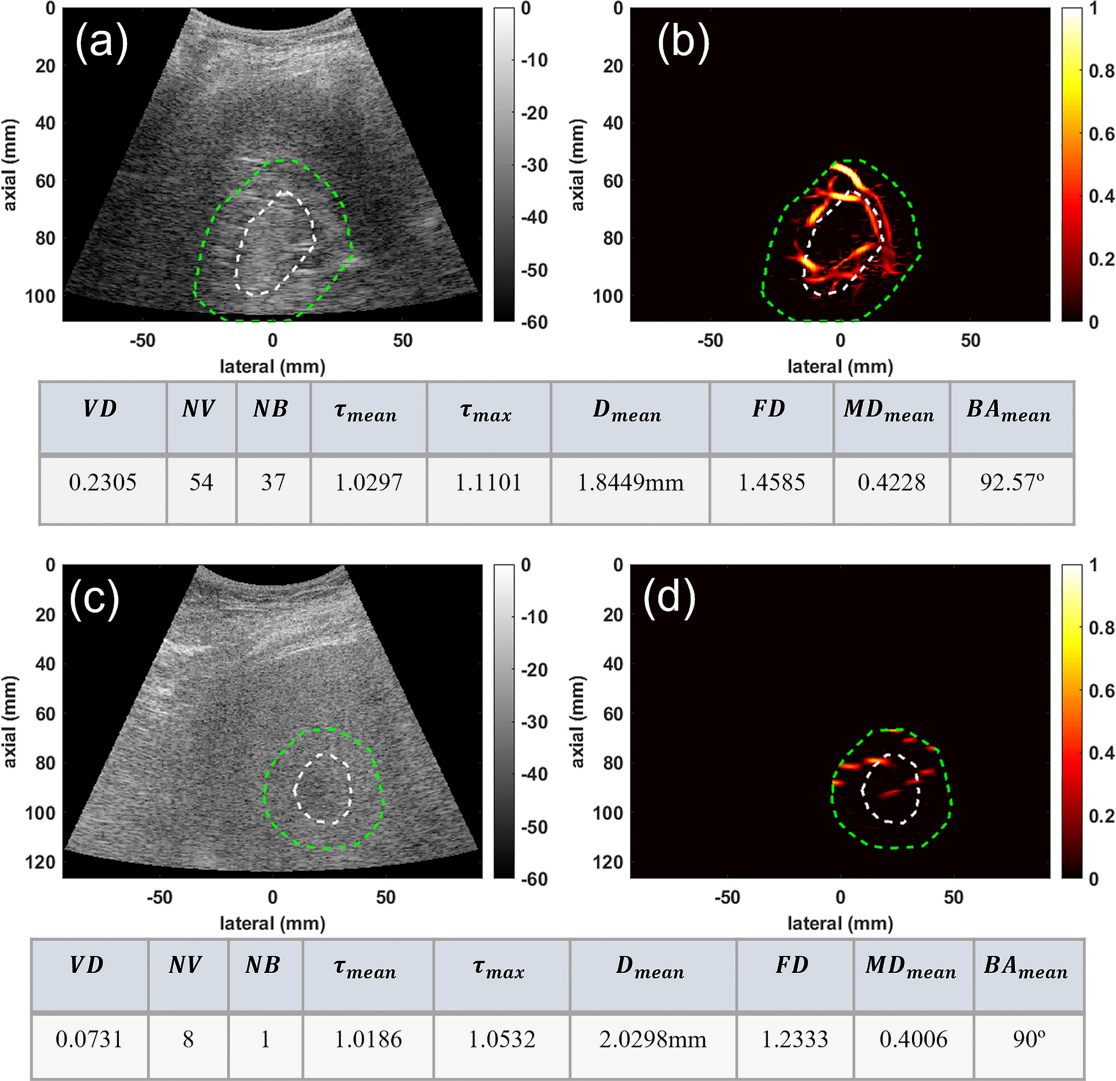
Visualization and quantification of deep-seated HCC and benign masses. **(A)** B-mode image of a deep-seated HCC tumor **(B)** Microvasculature image inside the dilated HCC tumor mask **(C)** B-mode image of a deep-seated benign lesion **(D)** Microvasculature image inside the dilated benign lesion mask. In all images the white dashed line indicates the boundaries of the lesion, and the green dashed line delineates the boundaries after a 10mm dilation. The tables below each row of images show metric values for the HCC (top) and benign (bottom) lesions.

In the second group, the conventional B-mode ultrasound and HMDI images of two deep-seated small liver masses, an HCC and a benign mass, are shown in **Figure 3** for visual comparison. This figure depicts the ability of HDMI in visualizing vasculature in and around deep-seated hepatic masses. The B-mode and microvasculature images of a dilated mass mask, centered approximately about 80mm, are shown in **Figures 3A, B**, respectively. This tumor is confirmed to be HCC. The B-mode and microvasculature images of another dilated mass mask, seated approximately about 90mm in depth, are shown in **Figures 3C, D**, respectively. This tumor is confirmed to be benign. The corresponding quantification metrics are shown in the tables below each row of images. The quantified metrics show higher values for *VD, NV, NB, FD, τ_mean_*, *τ_max_*, *MD_mean_*, and *BA_mean_* in HCC compared to the benign mass.

**Figure 4:**
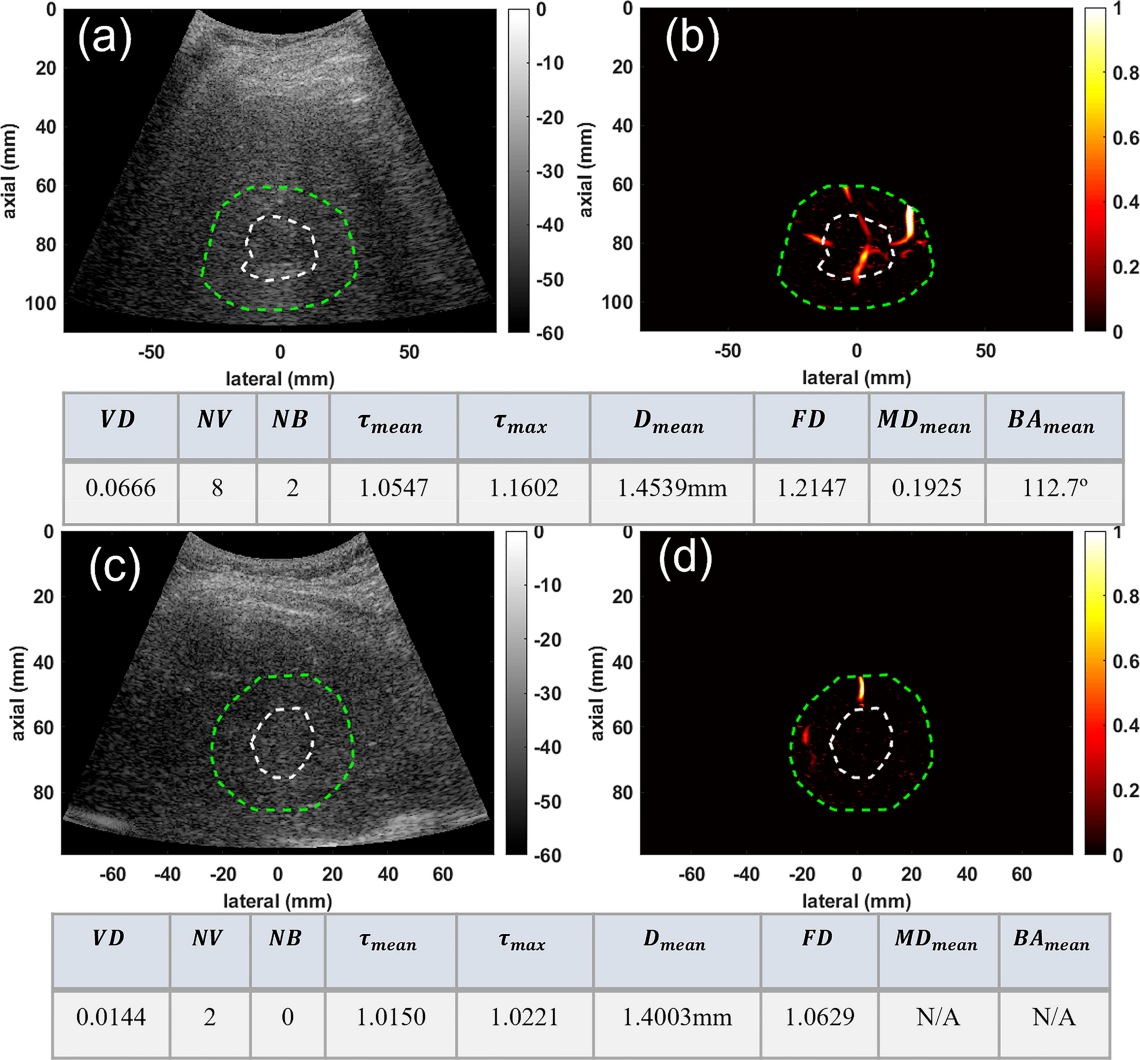
Visualization and quantification of small HCC and benign masses. **(A)** B-mode image of a 29mm HCC tumor **(B)** Microvasculature image inside the dilated HCC tumor mask **(C)** B-mode image of a 22mm benign lesion **(D)** Microvasculature image inside the dilated benign lesion mask. In all images the white dashed line indicates the boundaries of the lesion, and the green dashed line delineates the boundaries after a 10mm dilation. The tables below each row of images show metric values for the HCC (top) and benign (bottom) lesions.

We also demonstrated the potential of our method for characterization of very small liver masses in group three. **Figure 4** depicts the microvessel images of two very small masses under 3 and about 2cm, for a confirmed HCC and a benign mass, respectively. **Figures 4A, B**, respectively, show the B-mode and microvasculature images in and around an HCC tumor with an approximate size of 29mm in its largest axial/lateral dimension. Similarly, **Figures 4C, D** show the resulting images for a benign lesion with an approximate size of 22mm. The corresponding quantification metrics are shown in the tables below each row of images. The quantified metrics show higher values for *VD, NV, NB, FD, τ_mean_*, *τ_max_*, and *D_mean_* in HCC compared to the benign mass.

### 3.2 Differentiating malignant from benign liver masses

**Figure 5:**
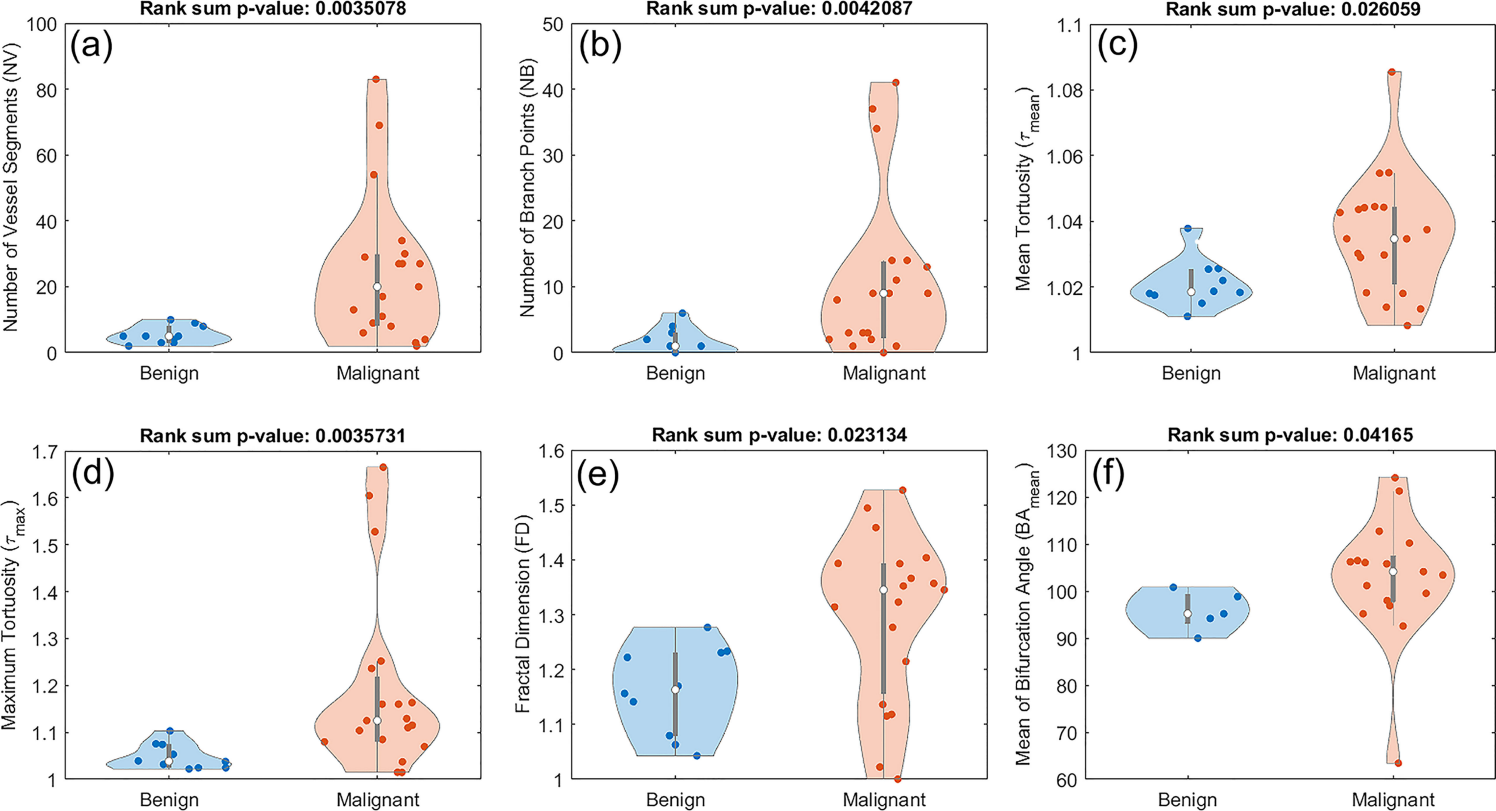
Composite boxplot/violin plots of distributions of features that exhibited statistically significant difference between benign and malignant lesions. **(A)** Number of vessel segments (*NV*) **(B)** Number of branch points (*NB*) **(C)** Mean tortuosity (*τ_mean_*) **(D)** Maximum tortuosity (*τ_max_*). **(E)** Fractal dimension **(***FD*) **(F)** Mean of bifurcation angle (*BA_mean_*).

Out of the nine examined features in this study, six of them showed a statistically significant difference between benign and malignant lesions. These features included number of vessel segments (*NV*), number of branch points (*NB*), mean tortuosity (*τ_mean_*), maximum tortuosity (*τ_max_*), fractal dimension (FD), and mean of bifurcation angle (*BA_mean_*). The corresponding p-values are 0.0035 for *NV*, 0.0042 for *NB*, 0.0260 for *τ_mean_*, 0.0035 for *τ_max_*, 0.0231 for *FD*, and 0.0416 for *BA_mean._* **Figure 5** shows the distribution of these metrics. All the violin plots in this figure and subsequent figures are generated in MATLAB 2019a using Bechtold’s “Violin Plots for Matlab” package (49). **Figure 5** shows composite boxplot/violin plots of distributions of features that exhibited statistically significant difference between benign and malignant lesions.

**Figure 6:**
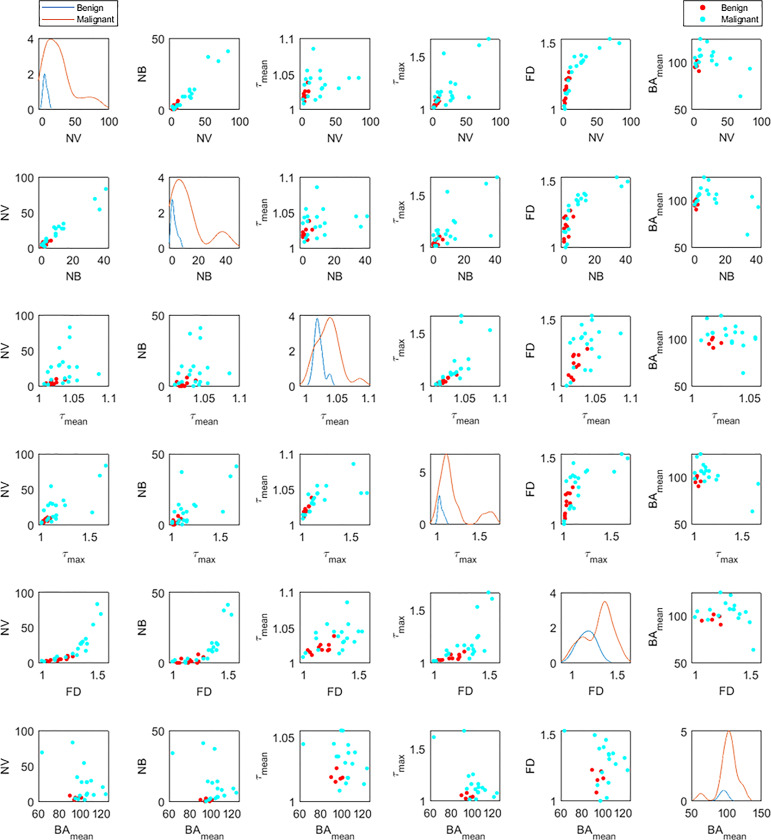
Distribution densities (diagonal) and bivariate scatterplots (off-diagonal) among statistically significant morphological variables for benign and malignant lesions. Variables include number of vessel segments (*NV*), number of branch points (*NB*), mean tortuosity (*τ_mean_*), maximum tortuosity (*τ_max_*), fractal dimension (*FD*), and mean of bifurcation angle (*BA_mean_*). For each distribution graph (diagonal figures), the orange line corresponds to the malignant lesions and the blue to the benign ones. In the off-diagonal scatterplots, the red dots correspond to the malignant lesions and the cyan to the benign ones.

**Figure 6** shows the distribution of the six significant features for benign (blue lines) and malignant (orange lines) cases (figures on the diagonal). These plots are also in agreement with the violin plots and show the distinctive potential of these features between the two groups. The off-diagonal scatterplots depict the correlation between each pair of features for malignant (red dots) and benign (cyan dots). There is a correlation between some features such as *NV*, *NB* and FD, or similarly between *τ_mean_* and *τ_max_*. However, a morphological feature such as bifurcation angle generally does not appear to be well-correlated with other metrics.

### 3.3 Differentiating HCC from benign liver masses

**Figure 7:**
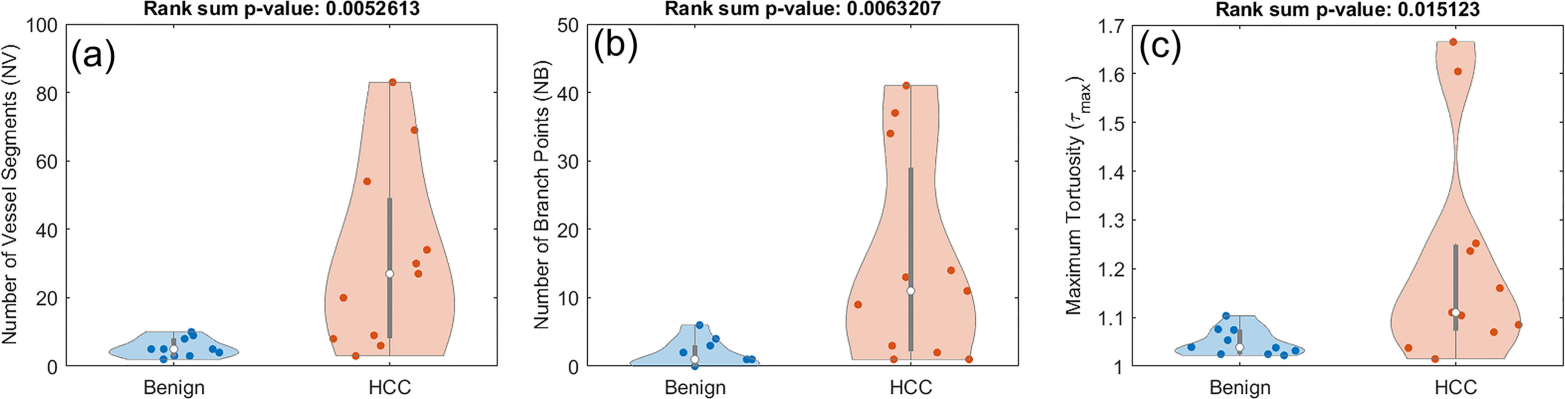
Composite boxplot/violin plots of distributions of features that exhibited statistically significant difference between benign lesions and HCC. **(A)** Number of vessel segments (*NV*) **(B)** Number of branch points (*NB*) **(C)** Maximum tortuosity (*τ_max_*).

In this section we investigate whether the pool of only HCC lesions have enough distinctive features when compared to benign lesions. Using the rank sum test for the new set of classes, only three of the features have significantly distinct distributions. These include *NV* with a p-value of 0.0052, *NB* with a p-value of 0.0063, and *τ_max_* with a p-value of 0.0151. The distribution of these three metrics for this analysis are presented in **Figure 7**.

The distributional summaries and analysis results of HDMI biomarkers for differentiation of benign and malignant as well as HCC and benign are detailed in **Table 2**.

**Table 2:** Associations of HDMI biomarkers for benign *vs*. malignant and benign *vs*. HCC.

HDMI biomarkers	Benign n=10	Malignant n=19	p value^a^
VD	0.045 ± 0.026	0.074 ± 0.058	0.242
D_mean_ (mm)	1.641 ± 0.356	1.555 ± 0.378	0.567
MD_mean_	0.350 ± 0.081	0.351 ± 0.101	0.815
FD	1.161 ± 0.080	1.295 ± 0.153	0.023
NB	1.700 ± 2.057	11.263 ± 12.520	0.005
NV	5.400 ± 2.716	24.894 ± 22.439	0.004
τ_mean_	1.020 ± 0.007	1.035 ± 0.018	0.027
τ_max_	1.048 ± 0.027	1.192 ± 0.192	0.004
BA_mean_	95.808 ± 4.214	102.783 ± 13.177	0.042
**HDMI biomarkers**	**Benign n=10**	**HCC n=11**	**p value^a^**
VD	0.045 ± 0.026	0.090 ± 0.070	0.149
D_mean_ (mm)	1.641 ± 0.356	1.667 ± 0.404	0.806
MD_mean_	0.350 ± 0.081	0.387 ± 0.106	0.595
FD	1.161 ± 0.080	1.312 ± 0.171	0.063
NB	1.700 ± 2.057	15.090 ± 15.109	0.007
NV	5.400 ± 2.716	31.181 ± 26.917	0.006
τ_mean_	1.020 ± 0.007	1.034 ± 0.015	0.063
τ_max_	1.048 ± 0.027	1.212 ± 0.221	0.016
BA_mean_	95.808 ± 4.214	105.553 ± 10.551	0.056

Data are presented as mean ± SD format ^a^p values are based on Wilcoxon rank sum test and a value less than 0.05 was considered statistically significant.

### 3.4 Morphological features in HCC compared to other malignancies

**Figure 8:**
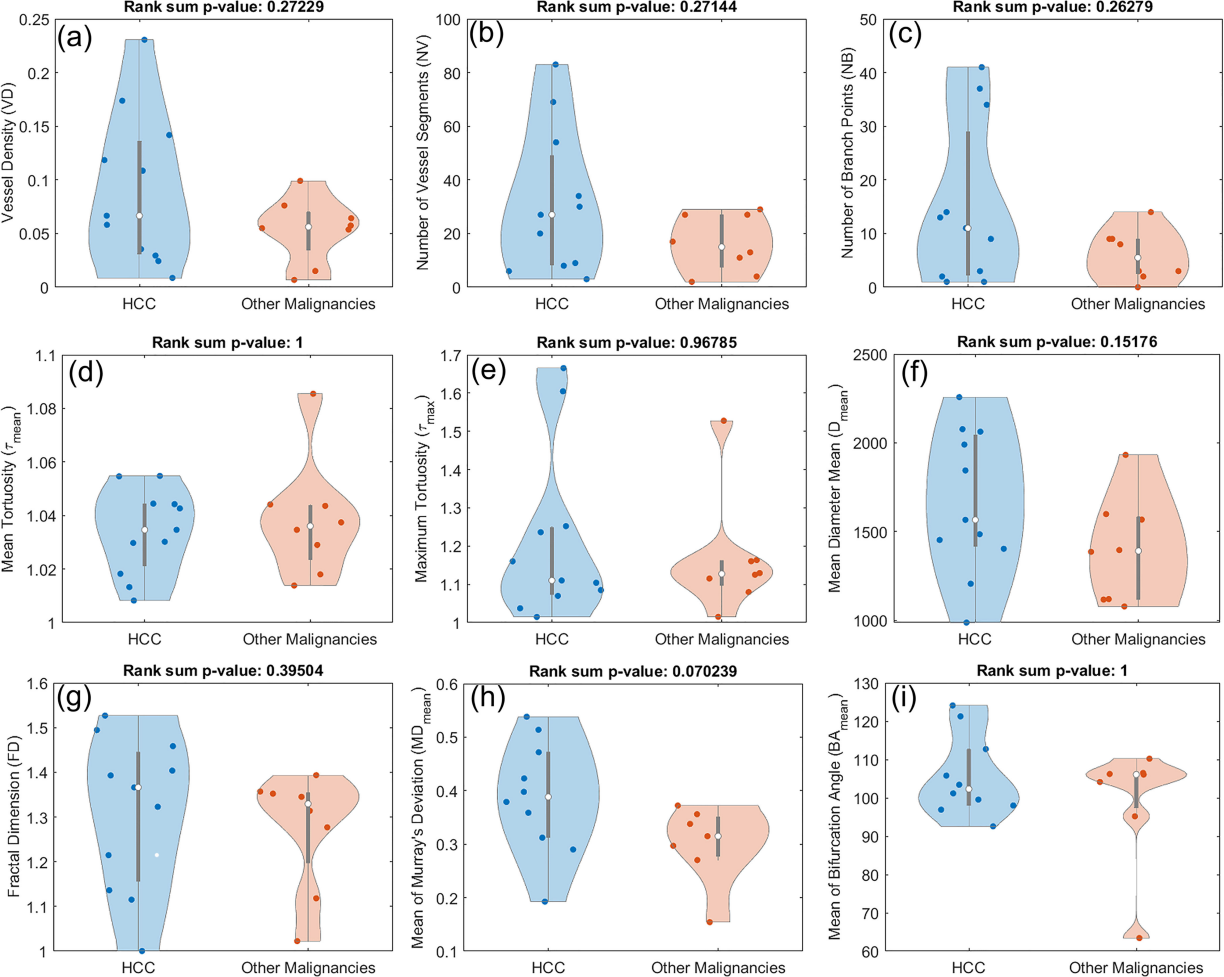
Composite boxplot/violin plots for all the utilized features in this study, comparing HCC with other malignant hepatic lesions (CCA and metastatic adenocarcinoma). None of the metrics proved distinctive between the two groups. **(A)** Vessel density (*VD*) **(B)** Number of vessel segments (*NV*) **(B)** Number of branch points (*NB*) **(D)** Mean tortuosity (*τ_mean_*) **(E)** Maximum tortuosity (*τ_max_*) **(F)** Mean of mean diameter (*D_mean_*) **(G)** Fractal dimension (*FD*) **(H)** Mean of Murray’s deviation (*MD_mean_*) **(I)** Mean of bifurcation angle (*BA_mean_*).

To examine whether any observable distinctions can be found in the morphological characteristics of the vasculature in HCCs and other malignancies, we performed a similar statistical analysis comparing the two groups. As shown in **Figure 8**, no statistically significant difference was observed in any of the features under consideration. However, some patterns can be seen in some of the metrics. For instance, higher medians and wider distributions including higher tails can be seen in HCCs for metrics such as *VD, NV,* and *NB*.

## 4 Discussion

This study investigated the discriminative potential of the quantitative biomarkers of contrast-free high-definition microvessel imaging (HDMI) for differentiating malignant and benign hepatic masses. Our findings show that HDMI biomarkers, *NV, NB, FD,* mean and maximum tortuosity (*τ_mean_* and *τ_max_*) as well as *BA_mean_* showed significant distinctions between malignant and benign lesion groups. The current study also found that *NV, NB,* and *τ_max_* have better discrimination performance than any other individual biomarker in differentiating HCC from benign. Another finding of this study is that HDMI biomarkers did not show differences between HCC and other types of liver malignancies, however, the numbers of patients are not enough to draw a statistically meaningful conclusion.

HCC is a highly vascular tumor in which angiogenesis plays a major role in tumor growth and metastasis, with vascular endothelial growth factor being a major player in angiogenesis (50). A hallmark of new vessel formation in tumors is their structural and functional abnormality. This leads to an abnormal tumor microenvironment characterized by low oxygen tension (51, 52). Few studies have proposed ultrasound microvessel imaging for differentiation of liver mases with (53) and without (41) contrast agents, limited to a pixel count method and visual inspection of images for the assessment of vessel shapes and distribution. The current quantitative HDMI study includes a wide range of tumor microvessel morphological biomarkers tested on a group of patients with liver masses and objectively discriminates benign and malignant liver tumors. A further advantage of the proposed method is that the enhancement and visualization of tumor microvessels at the submillimeter level can be done without the need for contrast agents.

This research explores the performance of *FD* as a new biomarker of tumor microvessels in contrast-free ultrasound microvessel imaging for differentiation of liver masses. In the current study, *FD* was found to have higher values in malignant compared to benign liver lesions. This finding is in agreement with the results of other studies, indicating that microvascular complexity calculated by *FD* may provide important diagnostic and prognostic information as well as insight into tumor angiogenesis (54, 55). A similar observation was also found for vessel tortuosity. The mean and maximum vessel tortuosity were significantly higher in the malignant lesions compared to the benign liver lesions. This indicates that vessel tortuosity metric can offer complementary objective information and may offer an additive value in discrimination when benign and malignant tumors are both hypervascular (56). The increased numbers of vessel segments and branch points and in our study indicate a higher level of vessel sprouting, recommending them as discriminators for benign and malignant liver tumors (57). Moreover, we observed less correlation between metrics such as *BA_mean_* with others, which indicates it is capturing a different set of characteristics of the vasculature and suggesting that the added information coming from such metrics can be beneficial in designing predictive models in our future studies.

Visualization and quantification of vascular structures inside cancerous lesions can be used as a tool for early detection of malignant tumors in different organs. The current study demonstrated the capability of this new quantitative method in capturing angiogeneses in very small liver lesions as small as 16mm, which is in keeping with the shown capability of this method for detecting angiogenesis in breast lesions as small as 4mm (47). Early or very early-stage HCC, determined as a single tumor with the largest diameter of the lesion measuring less than 3cm and 2cm, respectively, is a distinct clinical entity with a high rate of surgical cure (58). Ultrasound imaging without the use of contrast agents as a non-invasive and inexpensive imaging modality is the most commonly utilized means of surveillance and screening liver malignancies and HCC in particular (59). Technologies enabling microvasculature visualization using ultrasound devices can be beneficial in more accurate detection of hepatic tumors, particularly at their earlier stages. The current study also observed lower *VD, NV, NB, FD,* and vessel diameter in intrahepatic cholangiocarcinomas than HCC. This is in agreement with the literature indicating that intrahepatic cholangiocarcinoma (CCA) is hypovascular relative to HCC, probably due to its extensive stroma, and can be differentiated from HCC by imaging angiogenesis (60, 61).

Results obtained in this work as a feasibility study indicate that there is a potential for contrast-free ultrasound microvessel imaging with subsequent morphological analysis for detection of HCCs. There is limited published experience focusing on the use of ultrasound for quantitative vascular imaging in hepatic lesions. Most of such studies either make use of contrast agents for improved resolution and contrast in the resulting images or employ available devices and technologies for blood flow imaging with limited quantitative capabilities.

While patients with very early-stage HCC have excellent clinical outcomes, with 5-year survival rates over 60 to 80% after curative treatments (62–64), diagnosing cancer at a very early-stage is technically challenging. Diagnosis of early HCC often requires multiphasic cross-sectional imaging, typically dynamic contrast-enhanced MRI or CT. Both very early and early-stage tumors are relatively small, but both are hypervascular in the arterial phase, with specific imaging features that facilitate the diagnosis of HCC (65). In addition to the high costs of MRI and CT, the characterization of HCC requires the use of intravenous contrast with associated risks in those patients with underlying renal disease. Therefore, US-based imaging and microvessel morphology analysis of tumor using HDMI offer promise for noninvasive assessment of HCC. Moreover, early diagnosis would assist in optimal treatment planning and reduce the burden on healthcare costs. HCC detected at an early stage may be treated with surgical resection or percutaneous treatment, both of which are potentially curative and may reduce the mortality of HCC in patients with cirrhosis (66). Very early-stage hepatocellular carcinoma patients are deemed too early for liver transplantation, as such strategies as surgical resection and thermal ablation have gained popularity (67). Quantitative HDMI can potentially be employed for noninvasive assessment of HCC treatment schemes.

One limitation associated with this study is that the sample size was small and as a result, more sophisticated statistical analysis and development of predictive models were not tenable. In the future, we plan to apply this method on a larger cohort of participants including smaller liver lesions suspected of malignancy for more accurate evaluation of its performance for early detection of HCC. Also, there is a potential for data degradation due to breathing motions. In the future we plan to utilize and expand the motion correction and denoising algorithms (68–72) to reduce potential motion artifacts. Our future direction also includes the use of deep learning technique (73, 74) to mitigate artifacts resulting from undesirable sources of motion.

## 5 Conclusions

Accurate diagnosis of hepatic lesions is a challenging task and is of even greater significance when it comes to aggressive tumors such as HCC. The use of accessible and inexpensive methods for early detection of such tumors can greatly help with accelerating and streamlining the treatment process for patients. In this feasibility study, we investigated the use of a non-invasive contrast-free ultrasound microvasculature imaging technique for detection of malignant hepatic lesions and HCC in particular. By evaluating the morphological features of microvasculature visualized using this method, we were able to differentiate between the malignant and benign lesions. Results of this feasibility study indicate the potential of this technique. In the future, we will focus more on the detection of early-stage HCC as well as investigating the possibility of discriminating between different hepatic malignancies.

## Data availability statement

The raw data supporting the conclusions of this article will be made available by the authors, without undue reservation.

## Ethics statement

The studies involving human participants were reviewed and approved by Mayo Clinic Institutional Review Board. The patients/participants provided their written informed consent to participate in this study.

## Author contributions

Conceptualization, AA and MF. Methodology, AA and MF. Software, SS and RT. Validation, AA, MF, SS, NL, MO and TA. Formal Analysis, SS. Investigation, SS, RT, AA and MF. Resources AA and MF. Data Curation, SS and RT. Writing – SS. Writing – Review and Editing, SS, AA, MF, RT, NL, MO and TA. Visualization, SS and AA. Supervision, AA and MF. Project Administration, AA and MF. Funding Acquisition, AA and MF. All authors contributed to the article and approved the submitted version.
