# Supplementary material for: Morphometric analysis of tumor microvessels for detection of hepatocellular carcinoma using contrast-free ultrasound imaging: A feasibility study
Source: Front Oncol. 2023 Apr 13;13:1121664. doi: 10.3389/fonc.2023.1121664 (PMC10134399; doi:10.3389/fonc.2023.1121664)
Supplement: Supplementary file 1 [file DataSheet_1.docx]

Supplementary Material

Article Title

Soroosh Sabeti *, Redouane Ternifi, Nicholas B. Larson, Michael C. Olson, Thomas D. Atwell, Mostafa Fatemi, Azra Alizad*

*** Correspondence:** Azra Alizad: Alizad.azra@mayo.edu

# Supplementary Material:

Details of the quantitative HDMI methodology

The ultrasound microvasculature imaging and quantification scheme used in this paper comprises the following procedures: 1) a series of data and image processing steps to form enhanced images of the vascular networks within liver lesions; 2) a subsequent set of image processing algorithms to binarize and skeletonize these networks, segmenting the vascular structures and preparing them for quantitative analysis; and 3) quantification of the morphological features of the vessel segments. Details of these processes are presented as follows.

A) Microvasculature image generation and vessel segmentation

In order to create an image of the vascular structures inside hepatic lesions, a recently proposed multi-stage algorithm [1] was employed in this study. This approach has been named high-definition microvasculature imaging (HDMI) considering its ability to visualize microvessels as small as 300 µm. Visualization of these vessels is achievable through separation of strong tissue clutter signals from weak signals generated by slow blood flow within the vessels. Singular value decomposition (SVD) of the spatiotemporal ultrasound data and adaptive thresholding of the singular values/vectors allows us to remove the subspace corresponding to low-rank tissue clutter. Subsequent to SVD processing, morphological top-hat filtering followed by Hessian-based vessel enhancement is implemented on the power Doppler images generated from the clutter-filtered data.

Ultrasound data for processing were acquired using an ultrasound machine (Alpinion Ecube12-R, Alpinion Medical Systems, Seoul, South Korea), equipped with a curved array transducer C1-6 operating at 3.6 MHz frequency, in the plane-wave/high-frame-rate imaging mode. Post-processing on the acquired data was performed in MATLAB R2019a (The Mathworks Inc., Natick, MA, USA). SVD was performed on the data to separate out the tissue and blood subspaces. An automated analysis on the decay rate of the singular values (threshold-crossing in the second-order derivative of the singular value decay function) was done to define the rank of tissue clutter [1]. To reduce the inevitable background noise after SVD processing and enhance the visibility of vascular networks, we used a morphology-based background noise filtering (top-hat) in combination with a multi-scale Hessian-based vessel filtering to extract the structures with high “vesselness” characteristics. Following these procedures, the resulting microvasculature image was binarized using a global intensity threshold. Small structures that would potentially correspond to noise or artifacts from unwanted sources in the binarized image were removed. A region of interest (ROI) was manually chosen for each lesion based on the B-mode images in each data acquisition. The ROI underwent a 10mm dilation to ensure vascular structures in the periphery of the lesion are taken into account. The dilated ROI was then used to mask out signals/vessels in other parts of the image.

B) Quantification of microvessel morphological features

In order to prepare the image for quantification, the entire vascular network was skeletonized (using a thinning algorithm to find the mid-line of the vessels). Branch points were determined, dilated, and removed to determine individual vessel segments. At this step, parameters such as vessel density (VD), number of branch points (NB), and number of vessel segments (NV) could be computed. Other parameters, namely tortuosity (τ), fractal dimension (FD), Murray’s deviation (MD), and bifurcation angle (BA) were also evaluated, the description of which are briefly presented as follows:

1) Tortuosity (τ) in this study was estimated using the distance metric. The distance metric is defined as the ratio between the entire curve length and the length of the direct path between the two ends of a vessel segment.

2) Fractal dimension (FD) can be used as a metric for geometrical complexity of a structure. In this work the box-counting method was utilized to compute FD of lesional vascular structures.

3) Murray’s deviation (MD) is a measure of branching irregularities in vessel networks. A simple single bifurcation of a parent (mother) vessel generates two branch (daughter) vessels. Murray’s law indicates that to conserve the flow rate in the volume, the cube of the diameter of the mother vessel should match the sum of the cubes of the diameter of the daughter vessels. Any deviation from this assumption is captured by MD. In our analysis, at every bifurcation/branch point the vessel with the largest diameter was assumed to be the mother vessel.

4) Bifurcation angle (BA) is defined as the angle between two daughter vessels at a branch point. To estimate BA for each bifurcation, line fitting was applied to the daughter vessel curves, and the angle between them was computed.

More details on the different stages of quantitative HDMI processing can be found in [1-3].

References

1. Bayat, M., M. Fatemi, and A. Alizad, Background removal and vessel filtering of noncontrast ultrasound images of microvasculature. IEEE Transactions on Biomedical Engineering, 2018. 66(3): p. 831-842.

2. Ghavami, S., et al., Quantification of morphological features in non-contrast-enhanced ultrasound microvasculature imaging. IEEE Access, 2020. 8: p. 18925-18937.

3. Ternifi, R., et al., Quantitative biomarkers for cancer detection using contrast-free ultrasound high-definition microvessel imaging: fractal dimension, murray’s deviation, bifurcation angle & spatial vascularity pattern. IEEE transactions on medical imaging, 2021. 40(12): p. 3891-3900.
